# Paid sick days and stay-at-home behavior for influenza

**DOI:** 10.1371/journal.pone.0170698

**Published:** 2017-02-02

**Authors:** Kaitlin Piper, Ada Youk, A. Everette James, Supriya Kumar

**Affiliations:** 1 University of Pittsburgh, Pittsburgh, Pennsylvania, United States of America; 2 Department of Biostatistics, Graduate School of Public Health, University of Pittsburgh, Pittsburgh, Pennsylvania, United States of America; 3 Department of Health Policy and Management, Graduate School of Public Health, University of Pittsburgh, Pittsburgh, Pennsylvania, United States of America; 4 Department of Behavioral and Community Health Sciences, Graduate School of Public Health, University of Pittsburgh, Pittsburgh, Pennsylvania, United States of America; New York City Department of Health and Mental Hygiene, UNITED STATES

## Abstract

Access to paid sick days (PSD) differs by workplace size, race/ethnicity, gender, and income in the United States. It is not known to what extent decisions to stay home from work when sick with infectious illnesses such as influenza depend on PSD access, and whether access impacts certain demographic groups more than others. We examined demographic and workplace characteristics (including access to PSD) associated with employees’ decisions to stay home from work for their own or a child’s illness. Linking the 2009 Medical Expenditure Panel Survey (MEPS) consolidated data file to the medical conditions file, we used multivariate Poisson regression models with robust variance estimates to identify factors associated with missed work for an employee’s own or a child’s illness/injury, influenza-like-illness (ILI), and influenza. Controlling for gender, race/ethnicity, education, and income, access to PSD was associated with a higher probability of staying home for an employee’s own illness/injury, ILI, or influenza, and for a child’s illness/injury. Hispanic ethnicity was associated with a lower prevalence of staying home for the employee’s own or a child’s illness compared to non-Hispanic Whites. Access to PSD was associated with a significantly greater increase in the probability of staying home among Hispanics than among non-Hispanic Whites. Women had a significantly higher probability of staying home for their child’s illness compared to men, suggesting that women remain the primary caregivers for ill children. Our results indicate that PSD access is important to encourage employees to stay home from work when sick with ILI or influenza. Also, PSD access may be important to enable stay-at-home behavior among Hispanics. We conclude that access to PSD is likely to reduce the spread of disease in workplaces by increasing the rate at which sick employees stay home from work, and reduce the economic burden of staying home on minorities, women, and families.

## Introduction

People’s willingness to stay home from work and keep children away from school when ill is an important component of containing a contagious disease outbreak. For example, beginning with the 2009 H1N1 flu epidemic, the CDC recommended that people with influenza-like illness (ILI) stay home for an additional 24 hours after the fever subsides [1–3]. However, not everyone is able to adhere to these recommendations. One reason people may not engage in stay-at-home behavior is lack of access to paid sick days (PSD) [4–7].

In the United States (US), employees are not guaranteed access to PSD [8]. The US Bureau of Labor Statistics estimates that in 2015, 35% of employees did not have any access to PSD [9]. Employees who lack PSD are more likely to be low-income, and to work in smaller workplaces [10, 11]. Only 34% of employees in the lowest income groups had access to PSD compared to 89% of those in the highest income groups [9]. An analysis of the 2007 National Health Interview Survey showed that access to PSD differed by race/ethnicity, education, income, gender, occupational status, and self-reported health [12].

Employees without access to PSD were more likely to work while sick compared to those with PSD [12–14]. Compared to employed adults without PSD, those with PSD were shown to miss 1.5 more days of work for illness or injury [15]. PSD access has also been associated with employee health itself; for example, those with PSD had fewer injuries in the workplace than those without PSD [16]. Those without PSD were more likely to delay or forgo seeking medical care for their own or a family member’s injury or illness [15], and were more likely to seek care in an emergency department than in a doctor’s office [10, 12, 14]. Also, the risk of delaying medical care or seeking care in an emergency department was disproportionately higher in low-income groups without PSD [16].

Whereas previous studies have shown that PSD is associated with missing work for all-cause illness, little is known about the correlates of employees’ decisions to miss work when they are sick or their child is sick with an infectious illness such as influenza. Limited evidence suggests that for illness in general (not specifically influenza), PSD access may be associated with parental stay-at-home behavior when a child is sick. Approximately 60% of households with a child under 18y have two employed parents [17]: if there are no unemployed adults at home, parents have to decide if they should miss work and keep their sick child home from school [18]. Among low-income households, parents with PSD were more likely to stay home to care for an ill child than were parents without PSD [13] Researchers studying San Francisco’s PSD law [19] determined that parents with knowledge of paid sick days coverage were significantly less likely to report sending a sick child to school [20]. Also, the 2010 National Paid Sick Days Study found that parents without PSD were twice as likely to send a sick child to school or daycare compared to parents with access to PSD [14]. How PSD access and other demographic factors are associated with parental stay-at-home behavior when a child has influenza remains to be examined.

PSD may be especially important for working women if they are a single parent or more likely to stay home with an ill child than a male parent. Research conducted during the 1970s and the 1980s shows that women with young children were significantly more likely to miss work days than men [21, 22]. Every year since 1995, approximately 70% of women with children have participated in the labor force [23]. Yet, there are few current data available about gender differences in stay-at-home behavior for parents with an ill child. One recent analysis of the American time use survey 2011 showed that working women were more likely than men to report working when sick (i.e. engaging in presenteeism), and working when a child was sick [24]. The association between female gender and presenteeism was noted even among women with PSD access, suggesting that women may have a greater need for PSD than men [24].

Using computational simulations, universal access to PSD was shown to decrease influenza in the workplace by 6% [25]. However, important questions remain regarding how access to PSD may differentially impact demographic subgroups of employed adults, not only generally for any illness or injury, but also specifically for influenza-like illness (ILI). In this study, we use the 2009 Medical Expenditure Panel Survey (MEPS) consolidated data file along with the 2009 medical conditions file to examine employees’ probabilities of staying home from work for their own or their child’s illness, ILI, and influenza. We examine if demographic and economic factors, including race/ethnicity, gender, and household income affect employees’ decisions to stay home when ill or when a child is ill. Because the workplace environment may impact decisions independently of access to PSD, we also examine whether workplace size (number of employees) is associated with workers’ stay-at-home decisions. Our findings have implications for policy makers and risk communicators seeking to encourage sick people to engage in social distancing from work and school.

## Methods

The MEPS is an on-going, nationally representative survey that collects data on demographic factors, medical conditions, and health care usage from households across the US. The survey gathers data on each individual in participating households. The sample is drawn from a representative subsample of households that participated in the previous year’s National Health Interview Survey. MEPS features several rounds of interviews taking place over the course of 2 years [26]. For this study, we used data from 12,901 households collected during the 3 rounds of interviews in 2009.

From a total of 27,442 people 16y and older in the 2009 data file, we included those who were employed part-time (worked < = 34h per week) or full-time (worked >34h per week) during any of the three rounds of data collection in 2009. We excluded any respondents who were unemployed or self- employed in all three rounds of data collection.

Our outcomes of interest were whether or not an employee missed work for her/his own health condition, and whether or not an employee missed work for her/his child’s health condition. In each case, we examined whether an employee missed work for three health conditions: any illness/injury, ILI, and influenza. We chose to focus on the 2009 MEPS because the numbers of ILI and influenza cases were likely to have been higher due to the 2009 H1N1 pandemic compared to the following years. Using the medical conditions file, we coded ILI as ICD9 codes 79, 382, 460, 465, 466, 486, 487, 490, and 786 [27]. Influenza was coded as ICD9 code 487. The 2009 medical conditions file was linked to the 2009 full year consolidated data file using the person identifier.

Below we will describe our outcome and independent variables. To analyze the correlates of missing work for one’s own illness/injury, we used the outcome variable “DDNWRK” (days missed from work due to physical illness or injury, or a mental or emotional problem). We dichotomized the variable to examine stay-at-home behavior by coding no days missed as 0 and any days missed as 1. When examining stay-at-home behavior for ILI or influenza, we used “MISSWORK” (a flag associated with missed work for the associated health outcome—ILI or influenza) as the outcome variable. This variable was also coded as 0 if the respondent did not miss work for the associated medical condition, and 1 if she did.

To examine the probability of a worker missing work for a child’s illness/injury we used “OTHDYS” (days missed from work because of someone else's illness, injury, or health care needs) as our outcome variable. We dichotomized and coded this variable as 0 if the respondent missed no work and 1 if the respondent missed work to care for someone else’s illness/injury. Whether a worker missed work for each medical condition recorded for his/her children is not recorded in the survey. Hence, we examined whether PSD affected the probability of missing work for a household member’s illness/injury among parents whose children had ILI or influenza.

All analyses were conducted at the level of the employee. Independent variables for this study included access to PSD (“SICPAY”), as well as demographic and workplace-related characteristics, and self-rated health. An individual was considered to have PSD if they reported having paid sick leave when they were employed in any wave of the survey. Workplace size (“NUMEMP”), categorized as <50 employees or > = 50 employees, was based on data in round(s) when the individual was employed. Self-rated health (perceived health status (“RTHLTH”)) was categorized into good/very good/excellent, or fair/poor health. We categorized households as having at least 1 unemployed adult if a non-full-time student >16y age was unemployed in each of 3 rounds in the household. The respondent’s age, gender, household income, race/ethnicity, and educational level were included as independent variables. Race/ethnicity (coded based on “RACEX” and “RACETHNX” variables) was categorized as non-Hispanic White, non-Hispanic other, or Hispanic. Education (“EDUCYR”) was broken into 4 groups: less than a high school education, high school graduate, 1–3 years of college, or 4 or more years of college. Family income (“FAMINC09”) was divided into income quartiles so that quartile 1 represents the lowest income households.

We used complex survey analysis procedures to analyze the data in Stata version 14.0 [28]. These methods account for weighting and stratification of data (probability weight = PERWT09F, strata = VARSTR, and sampling unit = VARPSU) and provide robust variance estimates. Strata containing single units were scaled using the average of the variances from the strata with multiple units. Data were described using these survey estimation procedures, and a chi2 test was used to measure the relationship between each independent variable and PSD access. Because our outcomes of interest were not rare (for example, 58% of adults with PSD, and 40% of those without PSD missed work for their own ILI), we used Poisson regression with robust variance estimates rather than logistic regression [29, 30]. We conducted bivariate and multivariate Poisson regression analyses to measure the relationship between the independent variables and stay-at-home behavior. Using an F test, variables with a p value of <0.05 in bivariate analyses were included in multivariate models. Only respondents with no missing data for any independent variables were included. The total number of respondents for each outcome is shown in the tables. A Wald test was used to make comparisons between Poisson regression models. Given that PSD access differs by demographic factors, we wanted to examine if access to paid sick days modified the relationship between these factors and stay-at-home behavior. We tested interactions between race/ethnicity and PSD, gender and PSD, and income and PSD by adding them to the multivariate model. Additionally, smaller workplaces may discourage stay-at-home behavior due to the cost of each worker’s absence. To examine if such workplace norms had differential impacts on racial/ethnic minorities, we tested the interaction between race/ethnicity and workplace size.

To interpret the statistically significant interactions, we fitted models to a sample stratified by race/ethnicity. The regression coefficients of the stratified models were exponentiated to find the prevalence ratio of staying home.

Parsimony and conceptual considerations determined the parameters included in final models. In analyses of employee behavior for their own illness, income quartile and age were retained as control variables in all models. In analyses of employee behavior for their child’s illness, income quartile was included as a control variable in all models.

## Results

Our sample consisted of 12,044 employees over 16y age. The average age was 40.40 years (SD = 13.65). As shown in Table 1, 64% had access to PSD. Access to PSD was significantly associated with gender, race/ethnicity, income quartile, and education. Significantly higher proportions of employees in workplaces with > = 50 employees, and of those who reported good/very good/excellent health had access to PSD (Table 1). Table 2 presents the demographic characteristics of the 4,911 employees with children. In this sub-sample, 68% had PSD, and access varied by similar demographic and workplace characteristics as for the overall sample. A higher proportion of parents with no unemployed adults at home had access to PSD (Table 2).

**Table 1:** Characteristics of employees in sample (n = 12,044)^a^.

Variable	n	%^b^	% With PSD^b^
**Total PSD access**	7,225	64.33	-
Gender*			
Male	5,760	49.11	62.73
Female	6,284	50.89	65.87
Race/ethnicity*			
Non-Hispanic White	5,755	69.57	66.65
Non-Hispanic other	3,201	16.83	67.46
Hispanic	3,088	13.61	48.60
Education level*			
<High school	2,131	12.36	28.78
High school	3,542	27.93	56.19
Some college	2,990	26.10	66.07
4+ years college	3,381	33.61	82.81
Workplace size*			
<50	5,999	48.24	50.80
> = 50	6,045	51.76	76.93
General health status*			
Good/very good/excellent	11,011	92.60	64.95
Fair/Poor	1,033	7.40	56.58
**Employees with ILI**	3,455	30.66	65.14
**Employees with influenza**	485	4.09	57.97
	**Mean**^b^	**SD**	
Family income quartile*			
1 = lowest	$19,499	7,935	40.65
2	$42,973	6,941	61.82
3	$70,642	9,340	69.79
4 = highest	$139,365	51,624	76.90

PSD, paid sick days; ILI, influenza-like-illness; SD, standard deviation.

^a^ Sample includes all persons >/ = 16y who were employed (part time or full time) in 2009. Observations were dropped if missing any of the independent variables listed in the table.

^b^ Weighted using survey estimation procedures.

* Indicates p-value < 0.05 based on a chi2 test of the relationship between each variable and PSD access.

**Table 2:** Characteristics of employees with children (n = 4,911)^a^.

Variable	n	%^b^	% with PSD^b^
**Total PSD access**	3,027	68.41	-
Gender*			
Male	2,243	47.23	70.41
Female	2,668	52.77	66.62
Race/ethnicity*			
Non-Hispanic White	2,120	64.72	72.61
Non-Hispanic other	1,280	17.94	71.95
Hispanic	1,511	17.34	49.07
Education level*			
<High school	898	11.20	34.09
High school	1,426	26.82	58.57
Some college	1,192	25.60	72.33
4+ years college	1,395	36.38	83.45
Workplace size*			
<50	2,403	46.49	55.92
> = 50	2,508	53.51	79.25
Family structure*			
No unemployed adults at home	1,861	42.75	80.12
Has unemployed adults at home	3,050	57.25	59.66
**Employees with child with ILI**	2,698	57.18	67.98
**Employees with child with influenza**	425	9.28	66.40
	**Mean**^b^	**SD**	
Family income quartile*			
1 = lowest	$19,351	7,820	42.04
2	$43,220	6,621	60.43
3	$71,023	9,160	73.06
4 = highest	$138,932	49,813	83.95

PSD, paid sick days; ILI, influenza-like-illness; SD, standard deviation.

^a^ Sample includes all persons >/ = 16y who were employed (part time or full time) in 2009 with children. Observations were dropped if missing any of the independent variables listed in the table.

^b^ Weighted using survey estimation procedures.

* Indicates significance at 0.05 level based on a chi2 test of the relationship between each independent variable and PSD access.

### Association of PSD access with stay-at-home behavior for own health conditions

In a bivariate analysis (Column 2 of Table 3), adults with PSD had a higher probability of staying home for their own illness/injury compared to those without PSD (PR = 1.23, p<0.001). Not surprisingly, fair or poor self-rated health was associated with a higher probability of staying home from work compared to good/very good/excellent health (PR = 2.47, p<0.001). Controlling for self-rated health, income, age, and education in a multivariate Poisson regression model, PSD access remained associated with staying home from work for an illness/injury (APR = 1.26, p<0.001). Hispanics had a lower probability of staying home from work (APR = 0.78, p<0.001) compared to non-Hispanic Whites (Column 3 of Table 3), and those who were employed in workplaces with 50 employees or more had a higher probability of staying home from work for an illness/injury (APR = 1.08, p = 0.001) even after controlling for PSD access. Controlling for other factors, females had a higher probability of staying home from work for their own illness/injury (APR = 1.30, p<0.001) (Table 3). When added to this multivariate model, there was a statistically significant interaction between PSD access and race/ethnicity: PSD access was associated with a 39% increase in the probability of staying home for an employee’s own illness/injury among Hispanics and a 24% increase among non-Hispanic Whites. The interaction between race/ethnicity and workplace size was also statistically significant: employment in a larger workplace was associated with a 23% increase in the probability of staying home from work for an employee’s own illness/injury among Hispanics and a 4% increase among non-Hispanic Whites.

**Table 3:** Bivariate and multivariate analyses of stay-at-home behavior for own illness/injury, ILI, and influenza. Estimates are based on Poisson regression models with robust variance estimates.

	Illness/injury^a^		ILI^b^		Influenza^b^	
	n = 10,643		n = 3,455		n = 485	
	Bivariate	Multivariate	Bivariate	Multivariate	Bivariate	Multivariate
	PR (95% CI)	APR (95% CI)	PR (95% CI)	APR (95% CI)	PR (95% CI)	APR (95% CI)
**PSD access**						
No PSD	1.00	1.00	1.00	1.00	1.00	1.00
**Has PSD**	**1.23 (1.15–1.31)**	**1.26 (1.17–1.34)**	**1.42 (1.28–1.58)**	**1.42 (1.26–1.58)**	**1.42 (1.18–1.71)**	**1.29 (1.04–1.61)**
**Gender**						
Male	1.00	1.00	1.00	-	1.00	-
Female	**1.33 (1.26–1.40)**	**1.30 (1.23–1.37)**	1.07 (0.97–1.17)	-	1.20 (0.92–1.56)	-
**Income quartile**						
1 = lowest	1.00	1.00	1.00	1.00	1.00	1.00
2	0.95 (0.88–1.02)	**0.92 (0.86–0.98)**	1.08 (0.96–1.22)	0.99 (0.88–1.11)	1.18 (0.91–1.54)	1.07 (0.84–1.36)
3	0.93 (0.86–1.01)	**0.89 (0.82–0.96)**	1.03 (0.90–1.18)	0.92 (0.80–1.04)	**1.37 (1.12–1.68)**	**1.27 (1.04–1.55)**
4 = highest	**0.91 (0.84–0.97)**	**0.86 (0.80–0.93)**	1.02 (0.90–1.16)	0.89 (0.78–1.02)	1.08 (0.80–1.45)	0.93 (0.68–1.27)
**Race/ethnicity**						
Non-Hispanic White	1.00	1.00	1.00	1.00	1.00	1.00
Non-Hispanic other	**0.90 (0.85–0.96)**	**0.86 (0.81–0.92)**	0.98 (0.87–1.10)	0.95 (0.85–1.06)	0.93 (0.69–1.25)	0.89 (0.67–1.17)
Hispanic	**0.79 (0.73–0.85)**	**0.78 (0.73–0.84)**	**0.80 (0.71–0.91)**	**0.83 (0.74–0.93)**	**0.66 (0.48–0.92)**	**0.72 (0.53–0.98)**
**Education**						
< high school	1.00	-	1.00	1.00	1.00	1.00
High school	1.05 (0.96–1.16)	-	**1.28 (1.08–1.51)**	1.14 (0.96–1.34)	1.46 (1.07–2.01)	1.32 (0.94–1.84)
Some college	**1.13 (1.01–1.26)**	-	**1.26 (1.08–1.48)**	1.07 (0.92–1.26)	1.33 (0.87–2.02)	1.10 (0.73–1.66)
4+ years college	**1.13 (1.01–1.26)**	-	**1.29 (1.11–1.51)**	1.07 (0.90–1.26)	**1.67 (1.16–2.39)**	1.33 (0.89–1.98)
**Workplace size**						
<50	1.00	1.00	1.00	1.00	1.00	-
> = 50	**1.12 (1.06–1.17)**	**1.08 (1.04–1.13)**	**1.15 (1.05–1.25)**	1.06 (0.97–1.16)	1.12 (0.92–1.36)	-
**Health status**						
Good/excellent	1.00	1.00	1.00	-	1.00	-
Fair/poor	**1.47 (1.38–1.57)**	**1.51 (1.42–1.61)**	0.97 (0.83–1.12)	-	0.80 (0.58–1.11)	-
**Age**	**1.00 (0.99–1.00)**	**0.99 (0.99–1.00)**	1.00 (1.00–1.00)	1.00 (0.99–1.00)	1.00 (0.99–1.01)	1.00 (0.99–1.00)

^a^The outcome for illness/injury uses the entire 12,044-employee sample, constrained to those who have no missing data on any variables in the model.

^b^The outcome for ILI and influenza is constrained to those with an ILI or influenza diagnosis in order to ask if PSD access impacted stay-at-home behavior in those with medically confirmed ILI or influenza. For all outcomes, the number of employees (n) with no missing data for any independent variables is shown. ILI, influenza-like-illness; PR, prevalence ratio; APR, adjusted prevalence ratio; PSD, paid sick days. P-values <0.05 are shown in bold. Dashes indicate that variable was omitted from the multivariate model.

We examined stay-at-home behavior for an employee’s own ILI (Columns 4 and 5 of Table 3). Employees with PSD had a higher probability of staying home for their own ILI than those without PSD (APR = 1.42, p <0.001). Hispanics had a lower probability than non-Hispanic Whites of staying home from work for their own ILI (APR = 0.83, p = 0.002), controlling for age, income, and education. Gender, workplace size, and self-rated health were not significantly associated with staying home for ILI (Table 3).

Similar results were found when we examined stay-at-home behavior for an employee’s own influenza (Columns 6 and 7 of Table 3). Controlling for demographic factors, employees with PSD had a higher probability of staying home for their own influenza (APR = 1.29, p = 0.021). Hispanics had a lower prevalence of staying home from work when they themselves had influenza compared to non-Hispanic Whites even controlling for access to PSD, age, income, and education (APR = 0.72, p = 0.038). Gender, workplace size, and self-rated health were not significantly associated with staying home from work for an employee’s own influenza (Table 3). Interactions between race/ethnicity and PSD were not significant for the analyses examining stay-at-home behavior for an employee’s own ILI or influenza.

### Association of PSD access with stay-at-home behavior for child’s health conditions

Employees with children who had PSD had a higher prevalence of staying home from work for their child’s illness/injury compared to those who did not have PSD (PR = 1.39, p<0.001, Column 2 of Table 4). The association remained significant (APR = 1.24; p = 0.001) when controlling for gender, race/ethnicity, education, income, workplace size, as well as for having an unemployed adult at home. Females had a higher probability of staying home for a child’s illness/injury compared to males (APR = 1.46, p<0.001). Hispanics had a lower prevalence of staying home for a child’s illness/injury compared to non-Hispanic Whites (APR = 0.86, p = 0.019, Column 3 of Table 4). Controlling for the number of children at home did not impact the association between PSD and staying home from work for a child’s illness/injury (data not shown).

**Table 4:** Bivariate and multivariate analyses of stay-at-home behavior for child’s illness/injury, ILI, and influenza.

	Illness/injury^a^		ILI^b^		Influenza^b^	
	n = 4,409		n = 2,438		n = 387	
	Bivariate	MultivariateAPR (95% CI)	BivariatePR (95% CI)	MultivariateAPR (95% CI)	BivariatePR (95% CI)	MultivariateAPR (95% CI)
	PR (95% CI)	APR (95% CI)	PR (95% CI)	APR (95% CI)	PR (95% CI)	APR (95% CI)
**PSD access**						
No PSD	1.00	1.00	1.00	1.00	1.00	1.00
Has PSD	**1.39 (1.24–1.55)**	**1.24 (1.09–1.40)**	**1.27 (1.10–1.45)**	1.13 (0.98–1.31)	0.98 (0.68–1.42)	0.84 (0.58–1.21)
**Gender**						
Male	1.00	1.00	1.00	1.00	1.00	1.00
Female	**1.53 (1.38–1.69)**	**1.46 (1.32–1.61)**	**1.69 (1.51–1.89)**	**1.61 (1.43–1.80)**	**1.58 (1.22–2.05)**	**1.40 (1.08–1.83)**
**Income quartile**						
1 = lowest	1.00	1.00	1.00	1.00	1.00	1.00
2	0.94 (0.80–1.11)	**0.83 (0.71–0.98)**	1.01 (0.84–1.22)	0.92 (0.77–1.11)	1.45 (0.80–2.63)	1.31 (0.77–2.23)
3	0.98 (0.85–1.14)	**0.79 (0.68–0.92)**	0.90 (0.74–1.08)	**0.77 (0.64–0.93)**	1.26 (0.65–2.43)	1.12 (0.64–1.95)
4 = highest	**1.17 (1.03–1.32)**	**0.86 (0.74–1.00)**	**1.18 (1.02–1.36)**	0.92 (0.77–1.11)	1.26 (0.62–2.54)	1.04 (0.53–2.04)
**Race/ethnicity**						
Non-Hispanic White	1.00	1.00	1.00	1.00	1.00	-
Non-Hispanic other	**0.78 (0.69–0.88)**	**0.75 (0.66–0.85)**	0.90 (0.78–1.04)	**0.85 (0.74–0.98)**	0.85 (0.53–1.34)	-
Hispanic	**0.74 (0.66–0.83)**	**0.86 (0.75–0.97)**	**0.74 (0.63–0.86)**	**0.84 (0.72–0.99)**	0.78 (0.52–1.16)	-
**Education**						
< high school	1.00	1.00	1.00	1.00	1.00	-
High school	**1.26 (1.04–1.53)**	1.11 (0.90–1.36)	1.23 (0.95–1.59)	1.07 (0.83–1.38)	1.33 (0.70–2.53)	-
Some college	**1.59 (1.29–1.95)**	**1.32 (1.06–1.65)**	**1.44 (1.11–1.86)**	1.20 (0.92–1.55)	1.42 (0.74–2.70)	-
4+ years college	**1.73 (1.42–2.10)**	**1.41 (1.13–1.75)**	**1.58 (1.26–1.98)**	1.27 (0.99–1.63)	1.59 (0.94–2.68)	-
**Workplace size**						
<50	1.00	1.00	1.00	1.00	1.00	-
> = 50	**1.17 (1.06–1.30)**	1.07 (0.97–1.19)	**1.19 (1.06–1.34)**	1.11 (0.98–1.25)	0.82 (0.61–1.12)	-
**Family structure**						
No adults at home	1.00	1.00	1.00	1.00	1.00	1.00
Adults at home	**0.75 (0.67–0.84)**	**0.84 (0.75–0.93)**	**0.71 (0.62–0.82)**	**0.80 (0.70–0.92)**	**0.52 (0.37–0.72)**	**0.53 (0.37–0.77)**

^a^The outcome for illness/injury uses the entire 12,044-employee sample, constrained to those with a child and those who have no missing data on any variables in the model.

^b^The outcome for ILI and influenza is constrained to those with a child who had an ILI or influenza diagnosis in order to ask if PSD access impacted stay-at-home behavior in those whose child(ren) had medically confirmed ILI or influenza. For all outcomes, the number of employees (n) with no missing data for any independent variables is shown. ILI, influenza-like-illness; PR, prevalence ratio; APR, adjusted prevalence ratio; PSD, paid sick days. P-values <0.05 are shown in bold. Dashes indicate that variable was omitted from the multivariate model.

Column 4 of Table 4 shows that employed parents with PSD had a higher prevalence of staying home for a child’s ILI compared to parents without PSD (PR = 1.27, p = 0.001). However, after controlling for sociodemographic factors, workplace size, and the presence of an unemployed adult at home, PSD access was no longer significantly associated with parents’ stay-at-home behavior for a child’s ILI (APR = 1.13, p = 0.096). Women once again had a higher probability of staying home for their child’s ILI even after controlling for PSD access, education, income, race/ethnicity, workplace size, and family structure (APR = 1.61, p<0.001). Hispanics had a lower prevalence of staying home for a child’s ILI than non-Hispanic Whites (APR = 0.84, p = 0.035) (Column 5 of Table 4).

PSD access was not significantly associated with an employed parent’s stay-at-home behavior if a child had influenza (Columns 6 and 7 of Table 4). Women had a higher prevalence than men of staying home from work for a child’s influenza after controlling for PSD access, education, income, race/ethnicity, workplace size, and the presence of an unemployed adult at home (APR = 1.40, p = 0.013). Hispanics did not have a significantly lower prevalence of staying home compared to non-Hispanic Whites in this analysis. Interactions between race/ethnicity and PSD access, race/ethnicity and workplace size, income and PSD access, and gender and PSD access were not significantly associated with employee stay-at-home behavior for their child’s illness/injury, ILI, or influenza.

## Discussion

Staying home from work when ill, especially with infectious illnesses such as influenza, is an important health behavior and recommendation from the CDC [1–3]. By examining demographic and workplace characteristics associated with this behavior, our study identifies sub-groups of employees that face barriers to engaging in this recommended behavior, and highlights the continued role of employed women in providing care when a child is ill.

Our study adds to the growing body of literature highlighting the association of access to PSD with health behaviors [12, 13, 31]. Employees with PSD had a higher prevalence of staying home when ill compared to employees without PSD, reinforcing evidence that increasing access to PSD may encourage ill employees to stay away from work. This would reduce the burden of infection among employed adults [25], and potentially also reduce costs associated with ill employees working at lowered productivity [32].

### Association between race/ethnicity and stay-at-home behavior

We found ethnic disparities in access to PSD and in the ability to engage in stay-at-home behavior. Hispanics had a significantly lower prevalence of staying home when ill—for their own illness/injury, ILI, or influenza—than non-Hispanic Whites. PSD access was associated with a greater increase in prevalence of stay-at-home behavior among Hispanics compared to non-Hispanic Whites—for the employee’s own illness/injury. Thus, access to PSD may be important to enable stay-at-home behavior when ill, especially among Hispanics. Furthermore, we found that there was no significant interaction between PSD and ethnicity in stay-at-home behavior for an employee’s own ILI or influenza. Whereas PSD access was important to encourage stay-at-home behavior in general, and especially among Hispanics, other factors such as job security and workplace culture may have been additional important factors in stay-at-home behavior decisions during the 2009 influenza pandemic. During the 2009 H1N1 pandemic, which started in Mexico, Hispanics may have also been especially wary of repercussions for missing work with ILI, and may have hesitated staying home from work when ill with ILI even in the presence of PSD. Whether the association of PSD access with behavior overall and among Hispanics is similar during seasonal influenza epidemics should be examined in the future. In one nationally representative survey during the 2009 H1N1 influenza pandemic, Hispanics reported fewer resources at work than non-Hispanic Whites—including paid sick leave, job security, and the ability to work from home—to enable stay-at-home behavior when ill [33]. Furthermore, those without PSD were more likely to self-report influenza-like illness during the 2009 H1N1 influenza pandemic [6]. Employees are recommended to stay home when ill, especially with ILI, for 24 hours after symptoms subside [1–3]. Not everyone is able or willing to do this, however, due to lack of access to PSD, concern about job security or work not getting done in their absence, or because of a lack of workplace culture that encourages staying home when ill [34–36]. Constrained by data captured in MEPS, we were unable to directly examine whether factors such as job security or workplace culture were associated with stay-at-home behavior. Our study does provide some evidence that these factors should be examined in the future, as we discuss below.

### Workplace size and stay-at-home behavior

An indication that workplace culture may be important comes from our finding that employees in small workplaces (<50 employees) were less likely to stay home for their own illness/injury even after controlling for access to PSD. Furthermore, employment in a larger workplace was associated with a significantly greater increase in the probability of staying home from work for an employee’s own illness/injury among Hispanics than among non-Hispanic Whites. We conclude that small workplaces may need to actively promote healthy behaviors including staying home from work when ill by reducing real or perceived punitive reactions, and by communicating effectively with minorities.

### Working women and stay-at-home behavior for ill children

Two findings in our study point to the importance of PSD laws in reducing the economic burden of healthy behaviors in families. Women had a higher prevalence of staying home for a child’s illness/injury, ILI, or influenza than men even controlling for PSD access. This suggests that women are more often the primary caregivers for ill children. Somewhat surprisingly, we found that PSD access was not significantly associated with stay-at-home behavior for a child’s ILI or influenza. Medically attended ILI or influenza may have necessitated parental stay-at-home behavior irrespective of access to PSD. However, PSD access was similarly not associated with the higher reported levels of presenteeism behavior among women than men [24]. Taken together, this previous study and our current study show that women are more likely to report to work when ill or when a child is ill, as well as more likely than men to stay home to care for an ill child. Given the high rates of female employment in the US [37], these findings may point to a need for a greater number of paid sick days among women, and underline the importance of PSD policies that reduce the economic impact of gendered childcare behavior on families. PSD laws are thus important for women, children, and entire families in the workplace [11, 13, 38].

Our study has some limitations. The sample sizes for our three outcomes varied substantially. This could have resulted in statistical significance because of the large sample size, or at the other extreme, in not finding statistical significance due to low power for the smallest sample size. We were unable to examine the number of days of work missed for each health event because this information was not captured by MEPS. In addition, we used an overall measure capturing whether a parent missed work for a family member’s illness/injury in the absence of information about missed work specifically for a child’s illness. We constrained the dataset to employees with children to increase the likelihood that we are measuring employee stay-at-home behavior for a child’s illness rather than for another family-member’s illness. The ability to focus on stay-at-home behavior among families in which employees or children had ILI or influenza episodes during the 2009 H1N1 pandemic—using the 2009 MEPS medical conditions file—is, however, a strength of our study.

## Conclusions

In sum, we have shown that PSD access is associated with the CDC-recommended behavior of staying home from work when sick for an employee’s own illness/injury, ILI, or influenza. In addition, ethnic disparities exist in employees’ ability to engage in this behavior with Hispanics having a lower prevalence of staying home when ill, and possibly facing additional barriers to this health behavior in small workplaces. Females remain the primary caregivers for sick children. Though PSD access is not associated with parental stay-at-home behavior when children have ILI or influenza, access to this resource may lower the economic burden faced by families when a parent (often the mother) has to take time off from work to care for a sick child. Future studies should examine the costs and benefits of PSD laws to small and large workplaces and to society.
